# Nip and Snarler

**Published:** 1867-01

**Authors:** 


### NIP AND SNARLER.

IN resuming the editorial pen it was but natural that we should look around and see what new and useful appliances, in the way of labor saving, had been introduced. The most noticeable is the adoption, by The Dental Times, of a convenience we have long enjoyed in private, but never thought of applying to journalism, and, yet like the telegraph, it "is very simple when once found out."

We have a little dog, too small to be of much force, yet he can do as much barking as if he were twice as large. His name is Nip. The "Times" has one also. His name is Snarler. He calls his yelpings "Quarterly Notes." Nip barks oftener than quarterly. Nip is an English Terrier. Snarler's pedigree is "unknown." Nip's eyes were open before he was two weeks old. Snarler's are open now, one of his keepers tells us. Nip acknowledges two or three masters. Snarler recognizes five. Nip licks his masters' feet. Snarler beslavers his masters' books and institutions. Nip is very ferocious, but we soothe our friends by telling them he wont bite. Snarler appears cross too, and one of his keepers has kindly told us he wont bite. He tells us also, that Snarler is in a "distant city." By a singular coincidence, Nip is too, and not far from the same city. Nip has but one vice. Sometimes *he will lie*, to induce us to open the door that he may come in to the fire. I am sorry to find Snarler addicted to similar vices. Both should be ashamed of themselves, and leave such practices to the bipeds.

We congratulate the "Times," on being, in this improvement, ahead of all competition. W.

